# A Case of Thoracic Aortic Mural Thrombus and Multiple Hypercoagulable Etiologies

**DOI:** 10.7759/cureus.60949

**Published:** 2024-05-23

**Authors:** Jacob Wagner, Rebekah Lantz

**Affiliations:** 1 Department of Internal Medicine, Wright State University Boonshoft School of Medicine, Dayton, USA; 2 Department of Internal Medicine, Miami Valley Hospital, Dayton, USA

**Keywords:** mortality, malignancy, virchow's triad, myoproliferative disorder, incidentaloma, heterozygous factor v leiden, prothrombin gene mutation, hypercoagulability, thoracic aortic mural thrombus, tamt

## Abstract

Thoracic aortic mural thrombi (TAMT) are rare yet a significant cause of emboli and mortality. Hypercoagulability is thought to play a role in pathogenesis. A common association is prothrombin G20210A mutation. We present a case of an 87-year-old man with an incidentally found TAMT in the setting of prothrombin mutation, metastatic prostate cancer, and a myeloproliferative disorder. The patient had several causes activating Virchow’s hypercoagulability principle, contributing to a centrally located clot. Because of its paucity in the literature, we advocate for further research concerning treatment modalities of TAMTs as well as an additional and timely workup for hypercoagulable states to prevent further calamity.

## Introduction

Thoracic aortic mural thrombi (TAMT) are rare central clots that occur at an incidence of 0.5% in adults worldwide [1]. Although the mechanism for TAMT is not completely understood, Virchow’s classic triad of endothelial injury, hypercoagulability, and stasis of blood flow contribute to the pathogenesis of thrombus formation [2]. Underlying atherosclerosis of the thoracic aorta and hypercoagulability are thought to contribute. Atherosclerosis naturally progresses with age but can be mitigated with the early implementation of a healthy diet and statin therapy [3,4]. Hypercoagulable states of consideration include the prothrombin G20210A mutation, factor V Leiden, and underlying malignancy [4]. Recent trauma, surgery, or inflammation are less common contributors [5]. Specifically, the prothrombin G20210A mutation is present in 2-4% of the adult population and is increased in those of European descent [6].

Clinical sequelae including stroke, spinal cord ischemia, visceral infarction, and acute limb ischemia associated with TAMT are destructive [1,7]. Computed tomography (CT), echocardiography, magnetic resonance imaging (MRI), and angiography are modalities used to capture and diagnose this condition. Given the paucity of cases, there are no established guidelines for treatment. However, typical management involves therapeutic anticoagulation, medical thrombolysis, endovascular intervention, or surgical management [7].

We present a case of TAMT incidentally found on imaging in the setting of an underlying hypercoagulability mutation and malignancy. We emphasize a thorough hypercoagulable evaluation, for the clearest risk factor stratification and duration.

## Case presentation

**Figure 1:**
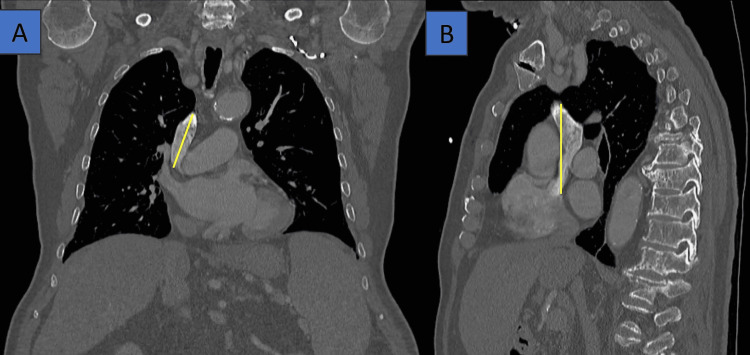
A lateral chest CT with contrast in the bone window. Yellow lines indicate a partially adherent thoracic mural thrombus in the (A) distal arch and (B) descending aorta

An 87-year-old Caucasian man presented to the emergency department (ED) after abnormal imaging of TAMT was noted during a diagnostic workup. He was a former smoker with a past medical history of hypertension, chronic lung disease, and prostate cancer. Prior to the ED, he was undergoing imaging and subsequent bone marrow biopsy (BMB) to differentiate lymphoma versus prostate cancer with axial metastasis. He complained of decreased appetite, weight loss, and diffuse pain but denied infectious symptoms or ill exposures. Specifically, the outpatient CT of the abdomen and pelvis with contrast revealed a partially adherent TAMT and possible atrial septal defect (ASD) (Figure 1). Additionally, there were multiple axial fractures of the thoracic and lumbar spine.

A hypercoagulable profile was performed, and therapeutic anticoagulation with a heparin drip was started. A transthoracic echocardiogram (TTE) was negative for ASD, indicating a cardioembolic source from a patent foramen ovale (PFO) was unlikely. The patient’s BMB was negative for lymphoma or other pathology, and a diagnosis of axial metastasis secondary to primary prostate cancer was presumed. Further, his hypercoagulable profile was heterozygous for a prothrombin G20210A gene mutation and elevated homocysteine level (Table 1) [4,8]. Subsequently, the hematology/oncology service recommended indefinite anticoagulation. In this unfortunate case, the patient was diagnosed with primary prostate cancer with axial metastasis, complicated by a hypercoagulable state due to a prothrombin G20210A gene mutation. This led to the development of a central clot in the thoracic aorta. Later, he was found to have a JAK2 mutation and was diagnosed with a myeloproliferative disorder. C KIT mutation was ordered but never completed to confirm systemic mastocytosis. He was prescribed hydroxyurea.

**Table 1:** Results of hypercoagulability workup APTT: activated partial thromboplastin clotting time; DRVVT: diluted Russell viper venom time: Ig: immunoglobulin; INR: international normalized ratio; LA: lupus anticoagulant (L) indicates lower than the reference range lab value. (H) indicates higher than the reference range lab value. Of note, the heparin was likely initiated prior to the timing of the hypercoagulable profile, schewing the aPTT result (false elevation).

Serum lab	Result	Reference value
DRVVT screen	64.5 (H)	30.1-44.1 sec
Kaolin clotting time	223.7 (H)	53.1-86.0 sec
Platelet count	295	140-440 K/μL
Prothrombin gene mutation	Detected	Not detected
Anti-thrombin III	79 (L)	80-120%
Protein C activity	74	70-130%
Plasminogen activity	91	80-120%
Activated protein C resistance	2.6	>2.0 ratio
Protein S, free	45 (L)	50-150%
Protein S, total	68	60-150%
Anti-prothrombin IgG	1	<20 G units
IgA, beta 2 glycoprotein	14	<20 A units
IgG, beta 2 glycoprotein	0	<20 G units
IgM, Beta 2 glycoprotein	5	<20 M units
Anticardiolipin, IgA	4	<22 APL
Anticardiolipin, IgG	13	<23 GPL
Anticardiolipin, IgM	1	<11 MPL
Phosphatidyl serine IgA	1	<20 APS
Phosphatidyl serine IgG	5	<16 GPS
Phosphatidyl serine IgM	4	<22 MPS
Homocysteine, total	34.6 (H)	6.0-16.8 mcmol/L
APTT LA screen	>200.0 (H)	30.8-44.8 sec
Thrombin time	>150.0 (H)	15.0-20.0 sec
Prothrombin time	16.8 (H)	11.7-13.9 sec
INR	1.4 (H)	0.9-1.1
APTT	>200.0 (H)	24.5-35.2 sec

The patient was discharged after a four-day hospitalization with cardiology and hematology services in consult. New medications were apixaban 5 mg twice daily and hydroxyurea 500 mg daily. After this time, he elected to follow up with the local Veterans Affairs, and there is no further data regarding his care or wellness after that time.

## Discussion

The formation of a TAMT in the absence of significant atherosclerosis is a rare yet potentially life-threatening condition. TAMTs are increasingly considered a source of peripheral embolization [7]. Only a few hundred cases are described in the literature. In the three decades from the 1950s to the 1980s, Machleder et al. noted 48 cases among 10,671 consecutive autopsies, accounting for an incidence of 0.45% [9]. Seventeen percent of these cases had evidence of distal embolization. Patients may present with chest pain, abdominal pain, or even asymptomatic pain [10-12]. However, the detrimental sequelae, such as acute ischemic strokes, abdominal infarction, and acute limb ischemia, are often the initial presenting signs of an aortic thrombus [1].

Thrombi can form along the ascending, descending, or arch of the aorta, though most develop in the proximal descending thoracic aorta (38%) [7]. TAMT may occur in a normal, undiseased aorta or in one with demonstrated atherosclerosis [5,7]. The underlying pathophysiology is multifactorial, but Virchow’s triad of thrombosis plays a role. Hypercoagulable states, heritable coagulopathies (e.g., factor V Leiden mutation, protein S deficiency, and antiphospholipid syndrome), infectious etiologies (e.g., sepsis, COVID-19), and solid tumor malignancies are all known to contribute to TAMT [4,13-16]. In an interesting case of TAMT with phospholipid syndrome, the patient was also infected with the novel SARS-CoV-19. This reflects that dual contributors may be present, and specifically that the viral sequelae of COVID-19 may unfold as an independent risk factor for central clot development [11].

In our case, the patient had a heterozygous positive prothrombin G20210A mutation, underlying malignancy, and advanced age with a calcified aorta. A prothrombin mutation alone is a known etiology of venous thromboembolic events in large US and Canadian cohorts [17,18]. However, the prothrombin mutation has not been described in association with a TAMT. Furthermore, the case is complicated by an underlying myeloproliferative disorder. It is known that myeloproliferative disorders increase the risk of venous thrombosis, but arterial thrombosis is rare [19]. Lower extremity ischemia secondary to arterial thrombosis with prothrombin gene mutation and myeloproliferative disorders has been established, but not central arterial thrombus [20,21]. The complex interaction between hypercoagulable states and thrombosis warrants additional investigation for coagulopathies, myeloproliferative disorders, and malignancies regarding their role in TAMT.

Because of its rarity, no definitive consensus on treatment exists, but there are suggestions for a new standard of care in the literature. Anticoagulation is indicated as first-line in nearly all cases, with the caveat that it does not completely eliminate risk, and medical management alone still carries a range of recurrence rates and persistent thrombus [1]. In certain instances, invasive approaches would need to be considered. Thrombolysis, thrombectomy, endovascular repair, aortic resection with reconstruction and grafting, and bypass have been described [1]. Open surgical approaches may be preferred in those with a substantial clot burden or a large, fragile thrombus, as an endovascular intervention has been associated with a high risk of embolization [1,15]. Minimally invasive approaches such as thoracic endovascular aortic repair (TEVAR) may also be considered in patients with failure of conservative measures, worsening clinical course, recurrent thrombus after surgery, or poor overall surgical candidacy. TEVAR was noted to successfully achieve thrombus resolution, regression, or exclusion in 80% of individuals but still carried a risk of subsequent embolization [7]. In patients with an underlying hypercoagulable state, both surgical and medical approaches have been utilized, but the general consensus is that at least a trial, if not indefinite, prescription of anticoagulation should entail endovascular, open surgical, and minimally invasive options reserved when conservative management fails [1,7].

This case demonstrates the complexity of thoracic aortic thrombi presentation, etiologies, and management. Furthermore, individuals with an incidental or symptomatic TAMT require investigation for underlying hypercoagulable contributions. Moreover, there is no published consensus for treatment, and shared decision-making should involve a multidisciplinary team of medical and surgical specialists.

## Conclusions

TAMTs are rare central arterial thrombi with a high risk of complications and mortality. Underlying causes of hypercoagulability should be investigated when arterial thrombus occurs. In our case, an elderly man was incidentally found to have a TAMT with multiple predisposing etiologies including prothrombin G20210A mutation, myeloproliferative disorder, and prostate cancer with axial metastasis. Anticoagulation is an effective first-line noninvasive treatment option, while endovascular or surgical approaches can be considered in certain cases alongside a multidisciplinary team of specialists. We emphasize a thorough evaluation investigating contributors to hypercoagulability including genetic mutations, inheritances, hyperproliferative disorders, and underlying malignancy to treat early and prevent devastating clinical sequelae.
